# Effect of mindfulness-based stress reduction in patients with acute myocardial infarction after successful primary percutaneous coronary intervention: a retrospective study

**DOI:** 10.1186/s12872-023-03346-0

**Published:** 2023-06-23

**Authors:** Jun-Jie Gu, Xiao-Shan Tong, Sha-Sha Meng, Shu-Hui Xu, Jin-Yan Huang

**Affiliations:** 1 Department of cardiology, Hangzhou First People’s Hospital, Zhejiang University School of Medicine, Hangzhou, 310006 Zhejiang China; 2 Operation room, Hangzhou Women’s Hospital, Hangzhou, 310016 Zhejiang China

**Keywords:** Mindfulness, Mindfulness-based stress reduction, Acute myocardial infarction, Primary percutaneous coronary intervention

## Abstract

**Objective:**

This study aimed to examine the effects of mindfulness-based stress reduction (MBSR) in patients with acute myocardial infarction (AMI) after primary percutaneous coronary intervention (PPCI).

**Methods:**

A retrospective study was conducted with data collected from AMI patients who underwent successful PPCI. The study included 61 cases that received 8-week MBSR intervention (MBSR group) and 61 cases that received weekly health education (control group) over the same period. Outcome measures, including hemodynamic parameters, psychosocial characteristics [Hospital Anxiety and Depression Scale (HADS), Perceived Stress Scale (PSS), Perceived Social Support Scale (PSSS)], health-related quality of life [HRQoL, 7-item Seattle Angina Questionnaire (SAQ-7)], and major adverse cardiovascular events (MACE), were assessed at baseline (T1), post-intervention (T2), 1 month after the post-intervention (T3) and 3 months after the post-intervention (T4).

**Results:**

Compared to the control group, the MBSR group showed improvements in blood pressure, specifically in systolic blood pressure (SBP) at T4, and diastolic blood pressure (DBP) at T3 and T4, and mean arterial blood pressure (MABP) at T3 and T4. Additionally, the MBSR group had lower scores of anxiety and perceived stress (HADS, PSS) and higher scores of perceived social support (PSSS) after the intervention. Furthermore, the MBSR group had higher scores on the SAQ-7 at all measurement points. The control group had a significantly higher total MACE rate compared to the MBSR group (26.23% vs. 9.84%).

**Conclusions:**

This study provides support for the potential benefits of MBSR as an adjunctive treatment for AMI patients undergoing PPCI.

## Introduction

Negative emotions such as depression, anxiety, and stress, are commonly experienced by cardiac patients following a myocardial infarction (MI), contributing to poorer mental health and increased risk for adverse outcomes [1, 2]. Primary percutaneous coronary intervention (PPCI), despite being a standard procedure for AMI, is still perceived as risky and stressful by patients due to nervousness, distress, and fear of the unknown [3–5]. Furthermore, psychopathologies like anxiety and depression are independent risk factors for cardiac morbidity and mortality [6]. Therefore, there is a need to identify psychological interventions that can alleviate negative emotions in AMI patients after PPCI.

Mindfulness, an popular psychological intervention worldwide originally designed in the 1970s by Jon Kabat-Zinn for chronic pain patients [5], involves cultivating conscious awareness and attention in the present moment, without judgment [7, 8]. One well-known form of mindfulness-based interventions (MBI) is the mindfulness-based stress reduction (MBSR) program, which consists of eight-week session aimed at reducing stress in clinical and non-clinical populations [9, 10]. The core elements of the program include mindfulness exercises during daily activities, such as body scanning, mental exercises, and physical exercises [7].

Over the past decade, several studies have investigated the effectiveness of the MBSR program in cardiac patients. Lundgren O et al. examined the feasibility of MBSR for CAD patients with depressive symptoms and found positive changes in depressive symptoms and mastery scores [11]. Another study conducted on male CHD patients demonstrated significant reductions in anxiety, depression, perceived stress, blood pressure, and BMI after receiving MBSR, with maintained therapeutic gains at the 3-month follow-up [12]. Additionally, a recent study explored the benefits of a modified MBSR intervention delivered via telephone for PCI patients experiencing psychological distress, highlighting the potential of this accessible approach in improving mental well-being [13]. Furthermore, a brief MBSR intervention was found to be beneficial for post-PCI patients younger than 60 years, aiming to reduce psychological symptoms of distress [14]. However, the specific contribution of MBSR in AMI patients after PPCI remains unclear.

Therefore, this study aimed to fill the research gap by conducting a robust evaluation of the effectiveness of MBSR in AMI patients after PPCI, providing valuable insights into the potential benefits of MBSR in this specific population. The study utilized a retrospective design and compares two groups: an MBSR group receiving 8 weekly sessions of 2.5 h each and a 1-day retreat, and a control group receiving weekly health education. Various outcome measures, including hemodynamic parameters, psychosocial characteristics, health-related quality of life (HRQoL), and major adverse cardiovascular events, are assessed at four time points. This study’s innovative approach and focus on the specific population of AMI patients after PPCI contribute to the existing literature and are highly relevant to post-AMI cardiac care.

## Methods

### Ethical statement

The study was approved by the ethics committee of our hospital, and the informed consent was waived due to the retrospective nature of the study.

### Study population

This retrospective analysis collected data between June 2019 and June 2022 on AMI patients who underwent successful PPCI, were moved to an internal medicine ward, and then discharged from our hospital. AMI was defined as acute symptoms of ischemia persisting for more than 30 min within 24 h from the onset of symptoms, with elevations in cardiac biomarker values (preferably cardiac troponin) and eventual detection of coronary artery disease by emergency coronary angiography [15]. The inclusion criteria were as follows: (1) aged ≥ 18 years; (2) successful PPCI, defined as final TIMI (Thrombolysis in Myocardial Infarction) flow grade 3 [16]; and (3) willingness to participate in all assessments. The study excluded patients with the following criteria: heart failure, valvular heart disease classified as more than moderate, moderate pulmonary hypertension, hypertrophic cardiomyopathy, significant renal impairment with an estimated glomerular filtration rate less than 15 mL/min/1.73 m^2^, attendance of mindfulness training or a stress reduction course (such as relaxation training or cognitive behavioral therapy) within the past two years, presence of psychosis, dementia or communication disturbances, and a history of alcoholism or other substance abuse.

### MBSR program

The study included 61 cases that received 8-week MBSR intervention (MBSR group) and 61 cases that received weekly health education (control group). Following a previously standard protocol [1, 17], the MBSR program lasted for 8 weeks and consisted of 8 weekly sessions, each lasting 2.5 h. Additionally, there was a full day of practice (6 h) in week 6. The program was facilitated by an experienced mindfulness trainer and scheduled 4 weeks after the PPCI procedure [18]. Participants were instructed to dedicate 45 min to formal homework exercises, which included mindfulness meditation, breathing practices, and gentle yoga. These exercises were to be completed on a minimum of 6 days per week, and participants were provided with taped audio instructions to guide their practice. As informal practices posed challenges in quantification, they were not specifically assigned. To ensure compliance with the homework requirements, a telephone-based monitoring system was employed on a weekly basis. The regular phone calls served as an opportunity for participants to report their progress, including the frequency and duration of their completed exercises. Outcome data were collected at 4 time points throughout the study: baseline (T1), post-intervention (T2), 1 month after the post-intervention (T3) and 3 months after the post-intervention (T4).

### Hemodynamic parameters

Impedance cardiography was used to measure hemodynamic parameters, including heart rate (HR), systolic blood pressure (SBP), diastolic blood pressure (DBP), and mean arterial blood pressure (MABP).

### Psychosocial characteristics

The perceived Stress Scale (PSS) and Perceived Social Support Scale (PSSS) were used to assess the degree of perceived stress and perceived social support from family, friends and significant others, respectively. The 14-item PSS questionnaire examined stress levels using a 4-point scale (score = 0: never; score = 4: very often), with a total score ranging from 0 to 56 [19]. The 12-item PSSS questionnaire was rated on a 7-point Likert scale (score = 1: strongly disagree; score = 7: strongly agree), with higher scores indicating higher levels of perceived support [20]. Levels of anxiety and depression were measured using the Hospital Anxiety and Depression Scale (HADS), which consists of subscales with a total possible score range of 0–21 each. The HADS uses a 4-point rating scale, with 0 representing “none” and 3 representing “severe”.

### Measurement of HRQoL

HRQoL was measured using the 7-item Seattle Angina Questionnaire (SAQ-7), which includes three domains: physical limitation, angina frequency, and quality of life [21]. Higher scores indicate better HRQoL.

### Clinical endpoints during the follow-up

Major adverse cardiovascular events (MACE) during the follow-up period were defined as heart failure (HF) requiring hospitalization, recurrent MI, repeat revascularization, and cardiac death. Hospitalization and death due to non-cardiac cause was not considered events.

### Calculation of sample size

A *post hoc* sample size calculation was performed using G*Power 3.1.9.2 software to determine the appropriate sample size for a *t*-test comparing the means of two independent groups. The calculation was based on the variable SBP at T4. The MBSR group consisted of 61 participants, with a mean SBP of 129.6 mmHg and a standard deviation (SD) of 11.61. The control group also comprised 61 participants, with a mean SBP of 135.9 mmHg and a SD of 12.83. The effect size (*d*) was determined to be 0.514. With an α error (significance level) set at 0.05, a *post hoc* sample size calculation was conducted, yielding a power of 80.53%.

### Statistical analysis

All statistical analyses were performed using Prism (version 8.0, GraphPad, San Diego, California, USA). Continuous data were summarized as means ± standard deviation (SD) or median ± interquartile range (IQR) after testing for normality using the Shapiro-Wilk test. Between-group comparisons were conducted using t-test or Mann-Whitney test, depending on the distribution of the data. Categorical variables were presented as n (%) and compared using chi-square or Fisher’s exact test. Two-tailed tests were performed for each comparison, with a *P* value of less than 0.05 considered statistically significant.

## Results

### Demographic and baseline characteristics

A total of 122 patients participated in this study, with a mean age of 58.69 ± 14.80 years (ranging from 18 to 72 years). The majority of the patients were male (68.03%), married (91.80%), and had a high school education or higher (68.85%). Table 1 provided detailed characteristics of the participants in the MBSR and control groups, showing no significant differences in gender (*P* = 0.244), age (*P* = 0.395), marital status (*P* = 0.788) and level of education (*P* = 0.320). Additionally, there were no significant differences in comorbidities, clinical presentation, lesion characteristics, culprit vessel, procedural characteristics, in-hospital adverse events, medication use at discharge, and length of hospital stay between the MBSR group and control group (all *P* > 0.05).

**Table 1:** Sample characteristics of included study participants

General characteristics	N	MBSR group (n = 61)	Control group (n = 61)	*P*
**Gender**				
Male	83	45	38	
Female	39	16	23	0.244
**Age (years)**				
< 60	59	17	12	
≥ 60	151	44	49	0.395
**Marital status**				
Single	3	2	1	
Widowed or separated	7	3	4	
Married	112	56	56	0.788
**Level of education**				
Below elementary school	18	8	10	
Middle school	20	9	11	
High school	53	24	29	
College or above	31	20	11	0.320
**Comorbidities**				
Hypertension	68	31	37	0.362
Diabetes mellitus	21	9	12	0.632
Previous stroke	1	0	1	1.000
Chronic obstructive pulmonary disease	6	2	4	0.680
Chronic kidney disease	1	1	0	1.000
Previous coronary artery bypass grafting	8	3	5	0.717
Previous congestive heart failure	3	2	1	1.000
Acute congestive heart failure	13	7	6	1.000
Cardiogenic shock	5	3	2	1.000
Ventricular arrhythmia	4	3	1	0.619
**Clinical presentation**				
STEMI	59	27	32	
Non-STEMI	63	34	29	0.469
**Lesion characteristics**				
One-vessel disease	59	30	29	
Two-vessel disease	37	19	18	
Three-vessel disease	26	12	14	0.906
**Culprit vessel**				
Left main coronary artery	12	7	5	
Left anterior descending artery	61	30	31	
Left circumflex artery	17	9	8	
Right coronary artery	32	15	17	0.912
**Procedural characteristics**				
Unfractionated heparin	115	59	56	0.439
Glycoprotein IIb/IIIa inhibitors	45	24	21	0.708
Intra-aortic balloon pump	9	6	3	0.491
PCI with stent	96	47	49	0.825
**In-hospital adverse events**				
Stroke	1	1	0	1.000
Acute renal failure	1	0	1	1.000
Acute vessel occlusion	1	0	1	1.000
Access site injury	1	1	0	1.000
**Medication use at discharge**				
Aspirin	121	61	60	1.000
Clopidogrel	82	40	42	0.847
Potent P2Y12 inhibitor	40	21	19	0.847
Dual therapy	120	60	60	1.000
Anticoagulant agent	3	1	2	1.000
β-blocker	104	51	53	0.799
Calcium channel blocker	7	4	3	1.000
RAS inhibitor	99	49	50	1.000
Statin	116	59	57	0.680
**Length of hospital stay (days)**		5 (3 ~ 7)	5 (3.25 ~ 8)	0.491

Note: Glycoprotein IIb/IIIa (GPIIb/IIIa); ST-segment elevation myocardial infarction (STEMI); “Length of hospital stay (days)” is presented as median with interquartile range (IQR)

### Comparison of hemodynamic parameters in AMI patients undergoing PPCI between MBSR and control groups

As shown in Table 2, there was no significant difference in HR between the MBSR and control groups at baseline and follow-up (all *P* > 0.05). However, the MBSR group showed decreased blood pressure compared to the control group at specific time points: SBP at T4 (*P* = 0.005), DBP at T3 (*P* = 0.018) and T4 (*P* < 0.001), and MABP at T3 (*P* = 0.003) and T4 (*P* < 0.001).

**Table 2:** Comparison of hemodynamic parameters in AMI patients undergoing PPCI between MBSR and control groups

	MBSR group (n = 61)	control group (n = 61)	*P*
HR (1/min)			
T1	79.54 ± 13.35	79.49 ± 11.29	0.983
T2	79.20 ± 12.64	79.54 ± 11.25	0.874
T3	79.44 ± 12.75	79.77 ± 11.71	0.883
T4	79.48 ± 12.49	80.23 ± 12.46	0.739
SBP (mmHg)			
T1	141.0 ± 19.00	142.0 ± 15.96	0.746
T2	137.3 ± 18.74	135.9 ± 12.88	0.637
T3	131.6 ± 11.59	135.9 ± 13.35	0.062
T4	129.6 ± 11.61	135.9 ± 12.83	0.005
DBP (mmHg)			
T1	77.74 ± 10.46	80.31 ± 8.047	0.130
T2	77.52 ± 10.61	80.26 ± 9.013	0.127
T3	75.92 ± 10.63	80.34 ± 9.714	0.018
T4	69.44 ± 7.522	80.00 ± 10.68	< 0.001
MABP (mmHg)			
T1	98.81 ± 9.550	100.90 ± 8.279	0.206
T2	97.44 ± 9.505	98.81 ± 7.771	0.387
T3	94.49 ± 7.884	98.86 ± 8.292	0.003
T4	89.50 ± 5.838	98.63 ± 8.535	< 0.001

Note: Acute myocardial infarction (AMI); Primary percutaneous coronary intervention (PPCI); Heart rate (HR); Systolic blood pressure (SBP); Diastolic blood pressure (DBP); Mean arterial blood pressure (MABP); baseline (T1), post-intervention (T2), 1 month after post-intervention (T3) and 3 months after post-intervention (T4)

### Effectiveness of MBSR intervention on psychosocial characteristics in AMI patients undergoing PPCI

**Fig. 1:**
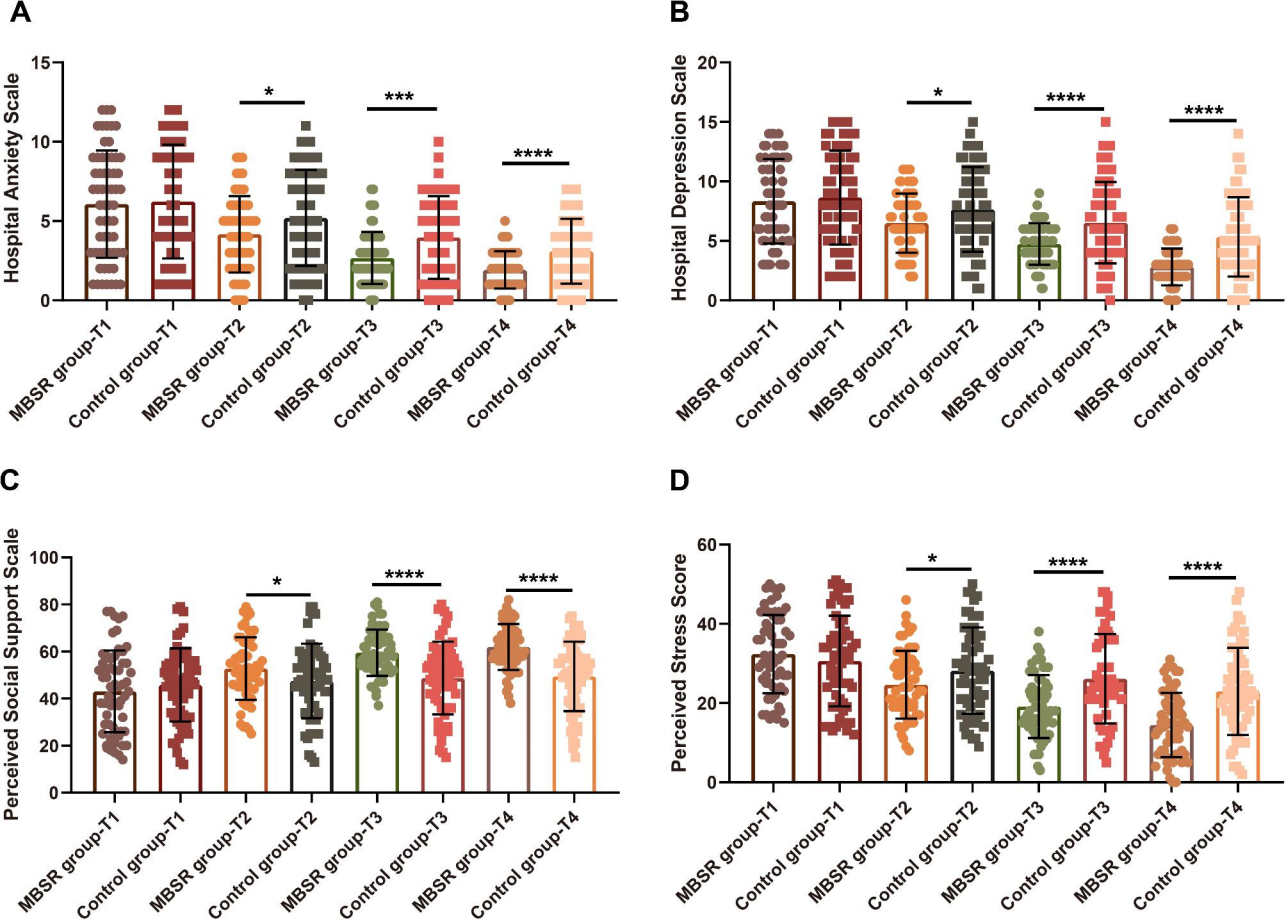
Effectiveness of MBSR intervention on psychosocial characteristics in patients with acute myocardial infarction (AMI) undergoing primary percutaneous coronary intervention (PPCI) Note: The psychosocial characteristics assessed include the Hospital Anxiety and Depression Scale (HADS, A-B), Perceived Social Support Scale (PSSS, C) and Perceived Stress Scale (PSS, D). The measurements were taken at baseline (T1), post-intervention (T2), 1 month after post-intervention (T3), and 3 months after post-intervention (T4). The significance levels are indicated as follows: * *P* < 0.05; *** *P* < 0.005; **** *P* < 0.001

Figure 1 illustrated a comparison of psychosocial characteristics between the two groups. Independent-sample t-tests revealed no differences in the scores of HADS, PSS and PSSS between the MBSR and control groups at baseline (T1) (all *P* > 0.05). However, significant differences were observed between the MBSR and control groups at T2, T3, and T4, with lower scores of HADS and PSS and a higher score of PSSS in the MBSR group compared to the control group (all *P* < 0.05).

### Improvement of MBSR intervention on HRQoL in AMI patients undergoing PPCI

**Fig. 2:**
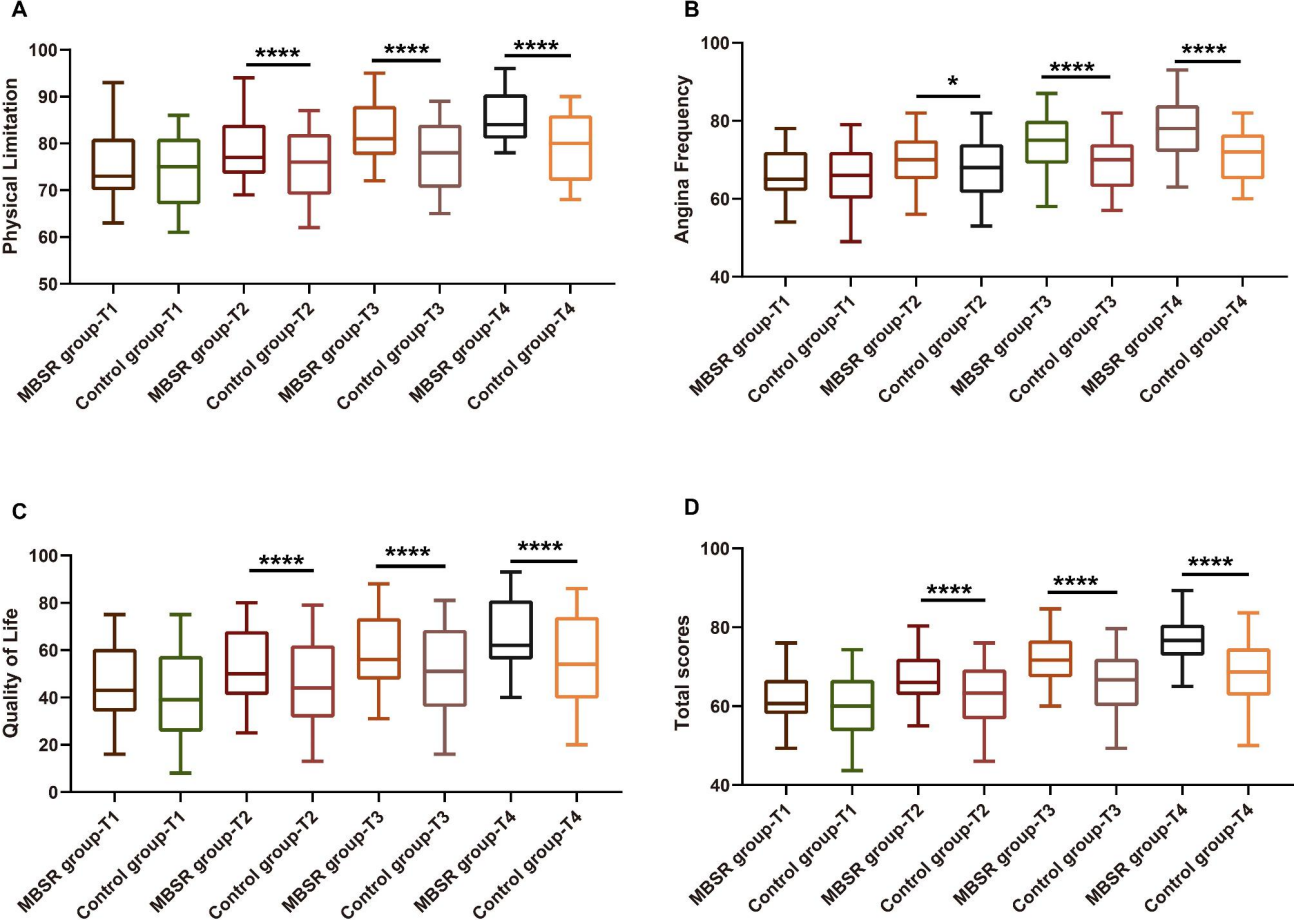
Improvement of MBSR intervention on Health-related quality of life (HRQoL) in patients with acute myocardial infarction (AMI) undergoing primary percutaneous coronary intervention (PPCI) Note: The comparison of HRQoL assessed by SAQ-7 score in AMI patients after PPCI between the 2 groups, including physical limitation **(A)**, angina frequency **(B)**, and quality of life **(C)** and total score **(D)**. The measurements were taken at baseline (T1), post-intervention (T2), 1 month after post-intervention (T3), and 3 months after post-intervention (T4). The significance levels are indicated as follows: * *P* < 0.05; **** *P* < 0.001

The independent-sample t-test with the SAQ-7 total score and its three domains showed no difference between the MBSR and control groups at baseline (all *P* > 0.05). However, significant improvements in physical limitation, angina frequency, and quality of life were observed in the MBSR group at T2-T4 compared to the control group (Fig. 2A-C). Furthermore, the overall SAQ-7 total score was higher in the MBSR group than the control group at all measurement points (Fig. 2D).

### Clinical endpoints in AMI patients undergoing PPCI after MBSR intervention

The occurrence of MACE was evaluated (Table 3). During the 3-month follow-up, 22 patients experienced MACE, including 11 cases of HF requiring hospitalization, 4 cases of recurrent MI, and 7 cases requiring repeated revascularization. No cardiac deaths were reported. Although a slightly lower proportions of AMI patients in the MBSR group developed heart failure requiring hospitalization, recurrent MI and repeated revascularization compared to the weekly health education group, the differences were not statistically significant (all *P* > 0.05). However, the total MACE rate was significantly higher in the control group as compared to the MBSR group (26.23% vs. 9.84%, *P* = 0.032).

**Table 3:** Clinical endpoints in AMI patients undergoing PPCI after MBSR intervention

MACE	MBSR group (n = 61)	control group (n = 61)	*P*
Heart failure requiring hospitalization	3 (4.92%)	8 (13.11%)	0.205
Recurrent MI	1 (1.64%)	3 (4.92%)	0.619
Repeated revascularization	2 (3.28%)	5 (8.20%)	0.439
Total	6 (9.84%)	16 (26.23%)	0.032

Note: major adverse cardiovascular events (MACE)

## Discussion

A growing body of research evidence suggested that MBSR has measurable and long-lasting benefits in medical and psychological well-being in various conditions [22, 23]. Consistent with previous literature, our study found that MBSR had positive effects on BP regulation. We observed a trend for improvement in SBP at the 3-month follow-up in the MBSR group, with decreased DBP and MABP at 1 month and 3 months post-intervention. These findings align with studies demonstrating the potential of in lowering BP in clinical populations such as cancer and hypertension [24, 25] as well as non-clinical populations like healthcare workers and victims of gun violence [26, 27]. Additionally, a previous study found sustained decreases in cholesterol levels after MBSR in older adults at risk for coronary artery disease [28], and which may further impact blood pressure regulation [29].

The ability of patients to track and cope with stress was also found to increase with greater awareness of their emotions and bodily sensations [5]. Our study demonstrated that AMI patients after PPCI who participated in the MBSR program showed improved psychosocial characteristics at post-intervention (T2), 1 month (T3), and 3 months (T4) follow-up compared to those who received weekly health education over the same period. The MBSR group exhibited lower perceived stress, anxiety and depression, as well as higher levels of perceived social support. These findings are consistent with previous studies reporting improved psychosocial characteristics following MBSR in cardiac patients. For example, Parswani M J et al. found the significant reductions in negative emotions and perceived stress in patients with coronary heart disease (CHD) who underwent an 8-week MBSR program, with therapeutic gains maintained at the 3-month follow-up [12]. Similarly, Lundgren O et al. demonstrated immediate and sustained improvement in depressive symptoms after an 8-week MBSR course in CAD patients following a coronary event [11]. These findings highlight the added benefits of using MBSR techniques to manage stress and psychosocial symptoms in cardiac patients, including those with AMI undergoing PPCI.

SAQ-19 was designed to be a more sensitive measure of recent changes in angina, and SAQ-7 was to assess patients’ satisfaction with the care they received for their coronary disease, which was more appropriate for most applications as reported by Thomas M et al. [30]. The MBSR group showed significant improvements in physical limitation, angina frequency, and overall QoL as measured by the SAQ-7 compared to the control group at all measurement points (T2-T4). These findings align with other studies examining the effects of MBSR on HRQoL. Jalali D et al. found stable HRQoL improvement in cardiovascular disease patients at the post-test and 3-month follow-up using the 36-item Short Form Survey (SF-36) after a MBSR program [31]. Nijjar et al. observed a similar trend in HRQoL between MBSR and control groups at 3 months in a pilot randomized controlled trial with cardiac patients eligible for cardiac rehabilitation [1]. These studies collectively demonstrated the effectiveness of MBSR in improving HRQoL. Finally, we evaluated the impact of MBSR on the occurrence of MACE. Despite having a small number of participants, our results revealed a significantly lower total MACE rate in the MBSR group compared to the control group (9.84% vs. 26.23%). This finding suggests that MBSR may have a positive effect on clinical endpoints in AMI patients after PPCI, which is an underexplored topic in the literature.

A major limitation of our study was its retrospective observational design instead of a randomized controlled trial. Furthermore, despite observing differences in blood pressure between the MBSR and control groups during the 3-month follow-up, it is crucial to acknowledge several limitations in our study. Firstly, the 3-month follow-up period may not fully capture the long-term effects of MBSR on blood pressure. It is possible that the observed differences might diminish or disappear beyond this time frame, as most AMI patients without complications tend to return to baseline or normal life and have stable hemodynamics after 3 months. Future studies with longer follow-up durations are needed to provide more comprehensive insights into the sustained effects of MBSR on blood pressure. Secondly, individual variations in response to MBSR could have influenced the observed differences in blood pressure. Some patients may have experienced more pronounced improvements, while others may not have shown significant changes. The influence of these individual differences should be considered when interpreting the results. It would be valuable to explore potential factors that may contribute to differential responses to MBSR, such as baseline characteristics or psychological factors. Lastly, it is essential to note that our study focused on a specific population of AMI patients without complications. The generalizability of our findings to other populations or individuals with different clinical profiles should be approached with caution. Future research should aim to replicate these findings in larger and more diverse samples to determine the broader applicability of MBSR in different patient populations.

## Conclusion

In summary, this study provides evidence that MBSR can be a beneficial adjunctive treatment in AMI patients undergoing PPCI, as it shows positive effects on alleviating negative emotion and perceived stress, increasing perceived social support, and reducing MACE. However, it is important to recognize that these findings are specific to the studied population and caution should be exercised in extrapolating the results to all cardiac patients.

## Data Availability

The datasets used and/or analysed during the current study are available from the corresponding author on reasonable request.
